# An HIV Protein Plays a Surprising Role in Gene Activation

**DOI:** 10.1371/journal.pbio.0030072

**Published:** 2005-02-08

Retroviruses are expert manipulators when it comes to co-opting their host's cellular resources. A great deal of human complexity stems from the vast repertoire of proteins and mechanisms dedicated to the business of regulating gene expression, and retroviruses like HIV have evolved myriad ways of redirecting that machinery to their own benefit.

Humans and other eukaryotes have three types of RNA polymerases, each charged with transcribing different types of genetic elements. RNA polymerase II transcribes protein-coding genes. RNA polymerases join with so-called general transcription factors to form a pre-initiation complex (PIC) on the gene's promoter, where it binds to region rich in thymine (T) and adenine (A) named the TATA box. The first transcription factor to associate with the TATA box is called TFIID, a large protein complex containing a protein that binds the TATA box (aptly named the TATA-box-binding protein, or TBP) and several cofactors called TBP-associated factors (TAFs). PIC assembly sometimes also requires activator proteins, which can enhance transcriptional activity by supporting proper elongation of nascent transcripts.

Tat, an activator encoded in the HIV genome, is required for HIV gene activation and viral replication. It affects these processes, the current model holds, by stimulating transcript elongation and increasing RNA polymerase's processing efficiency. In a new study, Tamal Raha, Grace Cheng, and Michael Green work with human cell lines and find evidence that Tat can also stimulate PIC assembly.

While most transcription factors bind to DNA, Tat binds to an area at the end of newly emerging viral RNA called the transactivation response element (TAR). Once bound, Tat recruits a cellular complex called P-TEFb (consisting of two subunits) to the HIV promoter, and enhances RNA polymerase's transcribing capacity. Previous studies in yeast had shown that activators appear to stimulate transcription complex assembly, leading the authors to ask whether Tat could play a similar role.

To study this question in living human cells, Green and colleagues turned to chromatin immunoprecipitation, a technique that detects proteins bound (directly or indirectly) to DNA. Working with three well-known effectors of transcription—an activator (Gal4-VP16), a transcriptional enhancer, and another viral activator called E1a—the authors show that what's true for yeast also holds for mammals, or at least for the human cell lines investigated here. Each effector was required for PIC assembly, which was in turn required to activate transcription.[Fig pbio-0030072-g001]

**Figure pbio-0030072-g001:**
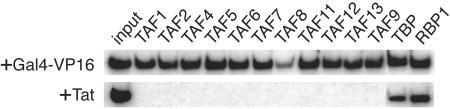
An unexpected mechanism of HIV-1 Tat action

The big surprise came in the next round of experiments, which explored Tat's influence on transcription and PIC assembly on the HIV promoter. As expected, transcription factors were “virtually undetectable” at the core promoter in the absence of Tat. Adding Tat recruited all the usual transcription factors to the promoter and increased transcription. But none of the TAFs that normally associate with TFIID were found. When the authors used the activator Gal4-VP16 to initiate HIV transcription, every one of the 11 TAFs studied appeared. None of them did so in the presence of Tat, suggesting that Tat-mediated HIV transcription doesn't rely on TAFs. Green and colleagues confirmed this hypothesis in experiments showing that Tat-driven transcription proceeded as usual in cells lacking TAFs. And they demonstrated that it is Tat—along with its cofactor P-TEFb, which is normally bound to RNA through Tat—that recruits the TAF-deficient TBP.

Altogether, these results show a surprising new role for Tat in stimulating assembly of a transcription complex. What's more, the complex lacks the TAFs typically linked to TBP in mammalian cells. Because their experiments analyzed only transcription complex assembly, the authors are careful to note that Tat may well stimulate assembly in addition to promoting transcription elongation. And it may be this resourcefulness that makes Tat such a potent activator—and HIV so hard to control. (For more on Tat's role in HIV transcription, see “Novel Enzyme Shows Potential as an Anti-HIV Target” [DOI: 10.1371/journal.pbio.0030074] and “A New Paradigm in Eukaryotic Biology: HIV Tat and the Control of Transcriptional Elongation” [DOI: 10.1371/journal.pbio.0030076].)

